# Polyaza functionalized graphene oxide nanomaterial based sensor for *Escherichia coli* detection in water matrices

**DOI:** 10.1038/s41598-021-96539-6

**Published:** 2021-08-19

**Authors:** Lina Rose, X. Anitha Mary, I. Johnson, Ganesh Srinivasan, Lakshmi Priya, Jebasingh Bhagavathsingh

**Affiliations:** 1 Department of Biomedical Engineering, Karunya Institute of Technology and Sciences, Coimbatore, Tamil Nadu 641 114 India; 2 Department of Robotics Engineering, Karunya Institute of Technology and Sciences, Coimbatore, Tamil Nadu 641 114 India; 3 Department of Millets, Tamil Nadu Agricultural University, Coimbatore, Tamil Nadu 641003 India; 4 Department of Applied Chemistry, Karunya Institute of Technology and Sciences, Coimbatore, Tamil Nadu 641 114 India

**Keywords:** Environmental sciences, Chemistry, Nanoscience and technology

## Abstract

Water quality is widely discussed owing to its significance in public health due to the inability to access clean water. Waterborne diseases account for the presence of pathogens like *Escherichia coli* (*E. coli*) in drinking water in the environmental community. Owing to the rapid increase of such bacterial microorganisms, a cost-effective sensor setup has been developed. Herein, we demonstrate the amine-functionalized graphene oxide (*f*GO) based 2D nanomaterial used to graft *E. coli* on its surface. The comparative analysis of the deposition of nanosheets on the glass substrate and PDMS was executed. The impedance variations of GO-based nanosensor at various concentrations of *E. coli* were performed and their potential difference was recorded. It was observed that the impedance changes inversely with the bacterial concentrations and was fed to the Arduino microcontroller. The experimental setup was standardized for the range of 0.01 Hz to 100 kHz. The obtained analog data was programmed with a microcontroller and the bacterial concentration in colony-forming units was displayed. The real-time analysis showsthe low-level detection of *E. coli* in aquatic environments. Experiments were conducted using the developed nanosensor to test the efficiency in complex water matrices and whose behavior changes with various physical, chemical, and environmental factors.

## Introduction

Nanomaterial-enabled sensors have paved the way for the development of high efficient smart devices as sustainable systems in the areas of food, water, health, and the environment^1^. The technology of nanosensors has been a promising tool in the area of bacterial detection and the pandemic agents as the major contaminant in the aquatic environment. Recent studies show that the mortality rate in children under is mainly due to diarrhea and pneumonia as the water-borne disease^2^. These epidemics are considered the most common cause ofthe high mortality rate among children. As quoted in the literature, the unclean habitat in India makes the country a predominant victim among other countries. The prevalent source of bacterial diarrhea includes the infections caused by Shigella species, *E. coli* and Cholera toxins, and salmonella species^3^. The WHO has illustrated the waterborne contaminants and their ill effects, in which the most common is *Escherichia coli*^4^. As per the reports, *E. coli* is a pathogen with a high risk of health-related issues, which is more persistent in water, less resistant to chlorine, and highly infective^5^. Though harmless, certain strains of coliforms could threaten humans and wildlife, and the bacterium is found in the gastrointestinal tracts and feces of warm-blooded animals. This is released into the environment through fecal deposition and therebyentering the water bodies due to human activities and climatic factors. In normal conditions, the strains of *E. coli* are suppressed by the strong acids in the intestines during digestion. Yet, certain strains like O157:H7 are the common factors in inducing diarrhoeic *E. coli*, which absorb nutrients from the intestinal walls and produce needle-like structures causing toxins to cause diarrhoeic conditions^6^.

There are various methods to detect bacterial concentration in food, milk, and water samples. The biochemical tests in the detection of bacteria, Pour Plate Method and Culturing Method are the prevalent measurement techniques used^7^. The enzyme and non-enzyme linked immunoassays, polymerize chain reaction (PCR) tests, voltammetric and amperometric methods, and optical detection tests like spectroscopy and fluoroscopy were also used in later stages. However, in all these determination methods the factors which affect detection varied with the performance criteria for a specific test. The various factors that affect the efficiency of detection are bacterial concentration, reacting agents, antigen–antibody bond formation, the relative amount of sample usage, and detection time^8^. The time for detection of bacterial contamination in water is a major challenge in assuring the water quality of any region. To address the issue, the development of a 2D-nanomaterial-based sensor is an ideal choice for the rapid, precise, live detection of pathogen contaminants (in colony formation unit) in water and calibrates the detection limits to assure accuracy with the science and technology tool.

In this view,Graphene, an allotrope of carbon with two-dimensional hexagonal lattices could be used as a nanomaterial for the proposed applications as it is 2D-layered with a large surface area^9^. Recent researchers have enlightened the biocidal properties of graphene in the detection and destruction of harmful bacteria^10^. To overcome the limited usage of graphene, the functionalized graphene oxides are found to be an effective nanomaterial for grafting various pathogenic antigens. The rigorous process of oxidation, exfoliation, and vapor deposition alters the physical, electrochemical, mechanical, and thermal properties of these molecular compounds. Also, the amine-functionalized graphene oxide based on 2D nanomaterials possessed a high degree of interlayer *d*-spacing with a change in the stacking of GO structures^11^. The ease of synthesis and less toxic properties make the polydimethylsiloxane (PDMS) remarkably used for many commercial portable sensor fabricationsdue to its nature of optically clear, inert, non-toxic, silicone-based biocompatible, and recyclable polymer^12^. Morteza et. al.^13^ have established quality assurance and quality control procedures of the biosensors for near real-time monitoring of *E.coli* in drinking water. Govindasamy et.al.^14^ have reported the GO-SrWO_4_ nanocrystals incorporated GO sheets for the arsenic drug detections in food products.The screen printed GO-Mn_3_O_4_ microcubes based nanocomposite was developed as a modified electrode for the detection of low-level nitrates^15^. Wu et. al.^16^ have innovated the rapid enzyme-based detection technique of *E. coli* using a membrane-based approach. Chromogenic substrates were used to optimize the enzyme hydrolysis and its sample concentrations. The detection of *E. coli* from milk samples and in-situ monitoring involves the use of a microelectrode array without an *E. coli* growth medium was also demonstrated^17^.The monitoring of the bacterial concentration at the water treatment site was also established in lakes in Norway, the equipment itself has to be incorporated with the plant, for regular monitoring levels^18^. Wang et.al.^19^ have demonstrated the modified electrode of GO decorated ZnO nano-flowers for the quantification of 8-HDG in human urine samples. Besides, all the existing methods are offline, involves the collection and carrying of test samples to the laboratory where it is tested. Many detection techniques have been focused on the measurement of optical^20^, electrochemical^21^, andpiezoelectrics methods^22^. The embedded use of nanotechnology in wireless sensor networks results in theinnovative automated strategies in water quality analysis^23^. Mani et al.^24^ have reported the real-time analysis of the electrochemical sensor fabricated from GO-Co_3_O_4_ polyhedron nanocomposites for the detection of H_2_O_2_ assay with the detection limit of 15 nM.Encapsulation of CuFeS_2_ in the GO planes was developed for the detection of methyl parazoxone (MOX) in the vegetables. The non-enzymatic sensor was used for the real time analysis of the detection of MOX with the detection limit of 4.5 nM^25^. Herein we report the functionalized GO material-based nanosensor for the low-level detection of *E. coli* concentration in freshwater sources. The AD5933 impedance analyzer was used to standardize, with the frequency range of 0.01 Hz to 100 kHz. The obtained analog data from the functionalized GO-based nanosensor was programmed with a microcontroller and the bacterial concentration in colony-forming units (CFU) was displayed for real-time analysis. Literature manifests the formidable situation of the lakes alongside the Noyyal River across the Coimbatore district in Tamil Nadu, India. Instead of this, less than 10 lakes in the above region were considered for the contamination analysis^26^.

## Results and discussion

The electrical conductivity of carbon and its derivatives had a great impact on the development of nanosensors and nanomaterials. The enhanced interlayer *d*-spacing distance and the chemical properties of *sp*^2^-hybridized carbon make it even more useful for device fabrication^27^. The challenges of the expensive and tedious manufacturing process of monolayer graphene restrict its usage in many industrial applications. To prevail over these negative features, graphene oxide is used widely, which is easier and cheaper to manufacture than graphene as such.

**Figure 1:**
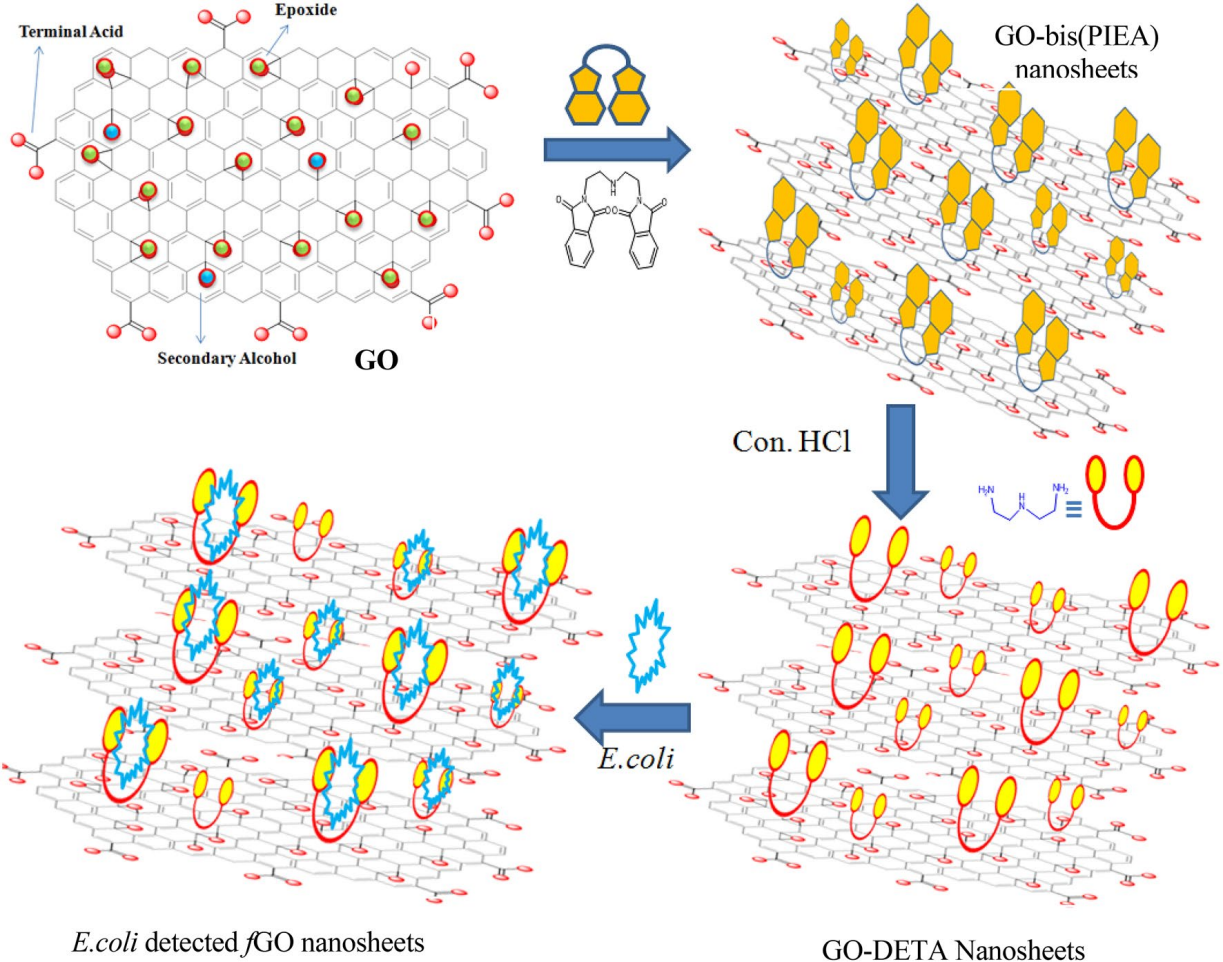
Schematic illustrations of amine functionalized GO for sensing application.

It also helps in enhancing physical and chemical properties like tensile strength, elasticity, conductivity, and so on. GO comprises three functional groups such as epoxide, alcohols, and terminal acids. The oxygenated functional groups of GO can be further modified to manipulate the properties of the graphene sheets. The amine-functionalized GO material was used to develop the sensor due to the enhancement of its *d*-interlayer spacing as shown in Fig. 1. The intercalated GO was identified for the sensing applications due to the enhanced heteroatoms on the surface which creates a significant room of coordination. The *f*GO material displays enhanced wettability and hydrophilic in nature, which eventually interacts the biological molecules with high polarity.Therefore, the efforts have been made to intercalatethe protected Diethylenetriamine (DETA) and subsequent deprotection strategy facilitates the covalent attachment of DETA, which contain heteroatoms such as nitrogen, and oxygen on the basal planes of GO. The unique morphology and textural properties are the key advantages of the covalently grafted GO. Particularly, grafting organic molecules on GO could lead to additional surface modification, resulting in the change of electronic properties.

Furthermore, covalent approaches offer the excessive benefit of attaining long-lasting stabilization of the isolated graphene sheets. The functionalization could improve the properties of GO such as surface charge, wettability and electron mobility, and hence conductivity. The developed intercalated GO nanosheets are used as the electrode material for the detection of pathological bacterial contaminants in water. On the other hand, *E. coli* is the most commonly found bacterial species, which produces Shiga toxins and it is gram-negative.

**Figure 2:**
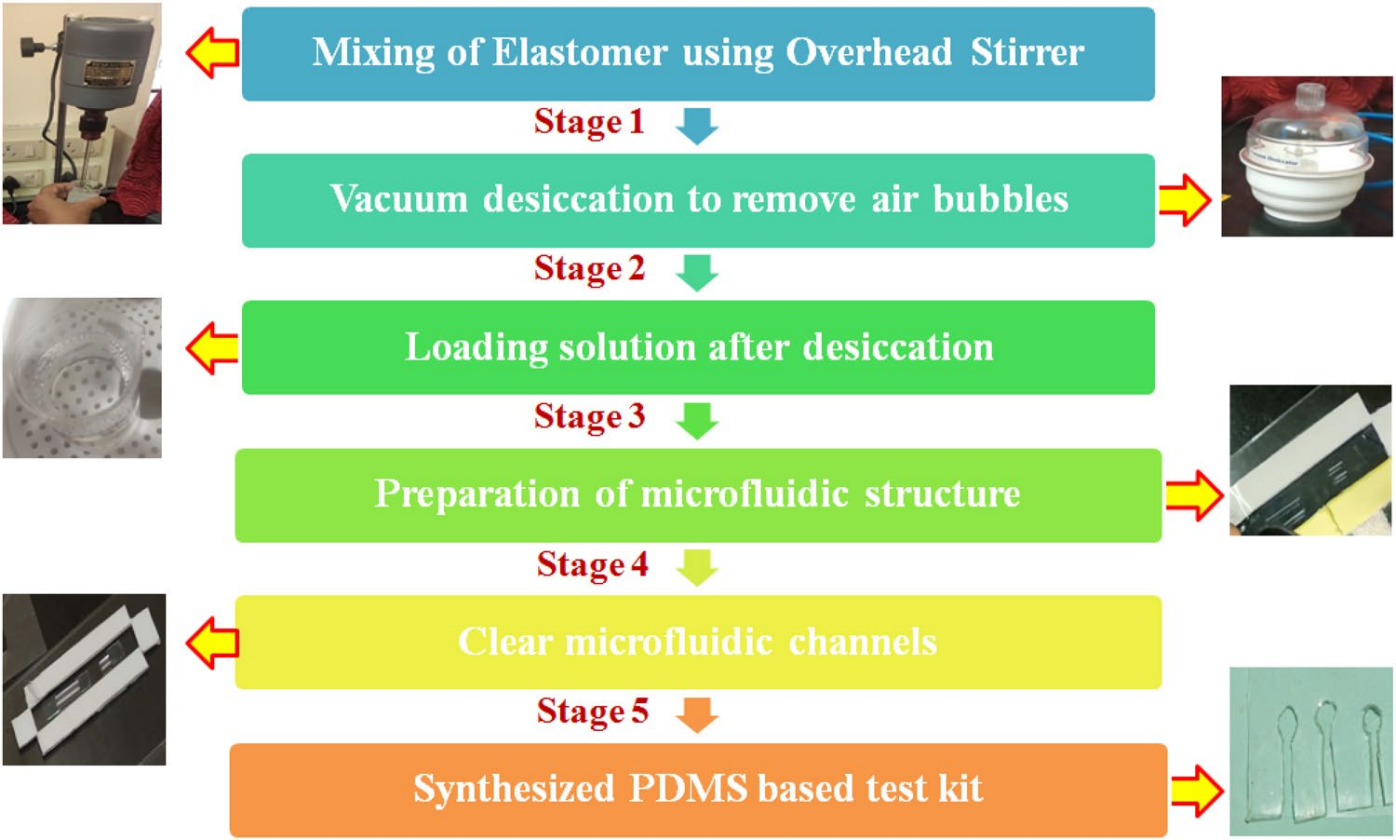
Process of development of PDMS-based test kit for the detection of *E. coli* in water quality analysis.

It causes illness and bacterial disorders, which are most detrimental to infants. The Heart-Brain Infusion (HBI) Broth was taken to culture *E. coli* samples (see Supplementary Fig. S10a–c).The stepwise device fabrication process of the nanosensor was displayed in Fig. 2.

**Figure 3:**
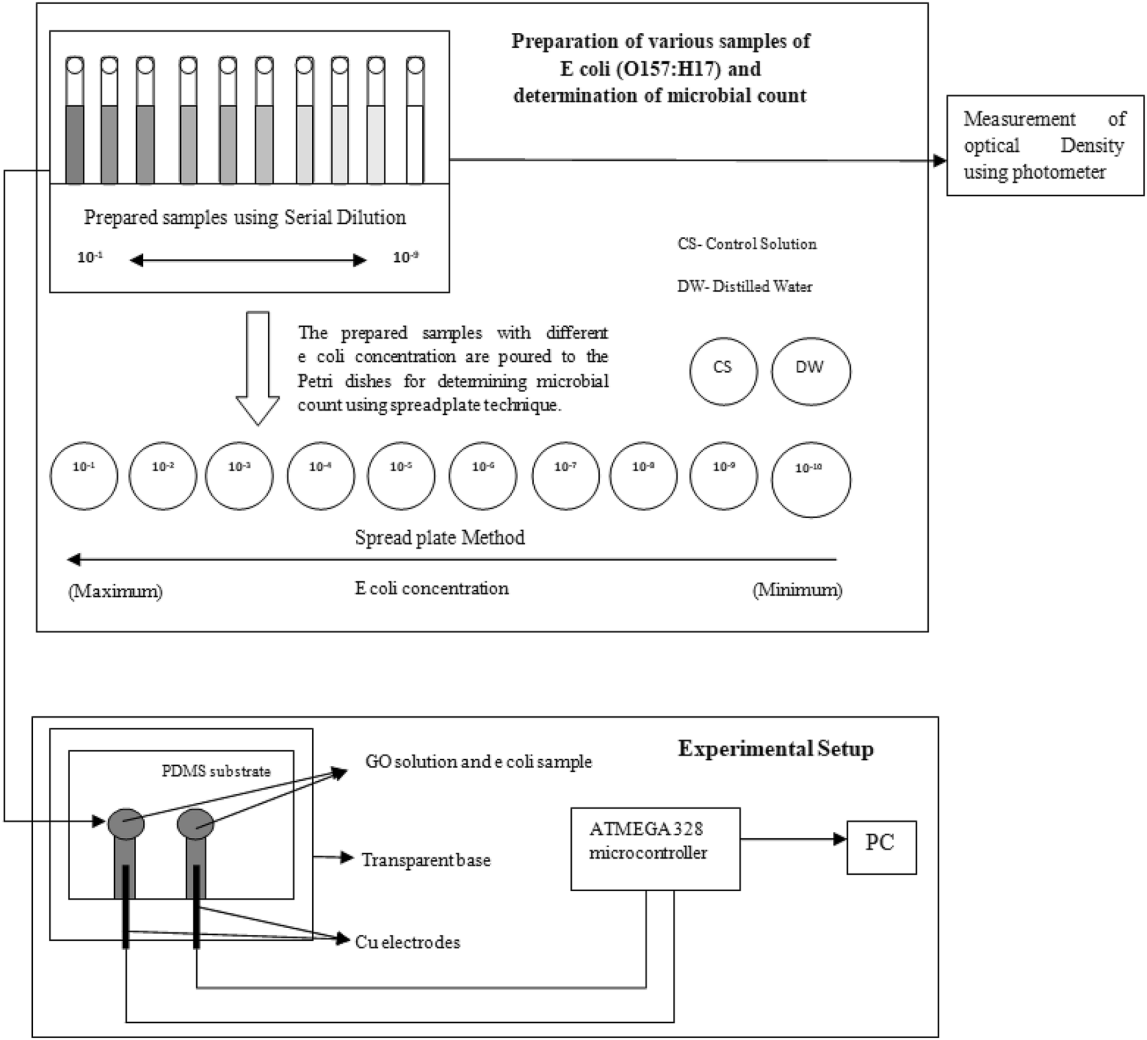
Outline of synthesis of bacteria, determination of microbial count, and nanosensor-based experimental setup.

The polymeric PDMS substrate was fabricated by blending with the curing agent using an overhead stirrer. The residue was desiccated overnight under a vacuum to remove the air bubbles.The resulting transparent loading fluid was transferred into the microfluidic template module and allowed to cure to get PDMS based test kit. The as-developed test kit was channelized with the functionalized GO and the lead for the sensing experiments.The experiment was performedoffline, using the developed nanosensor to measure the bacterial count/CFU. The procedure of the experiments underwent was illustrated in Fig. 3. The copper electrodes were connected to the analog pin of the microcontroller which reads the analog voltage. The inbuilt analog to digital converter in the controller produces step changes for each analog voltage value, which in turn was an indication of the concentration change.

**Figure 4:**
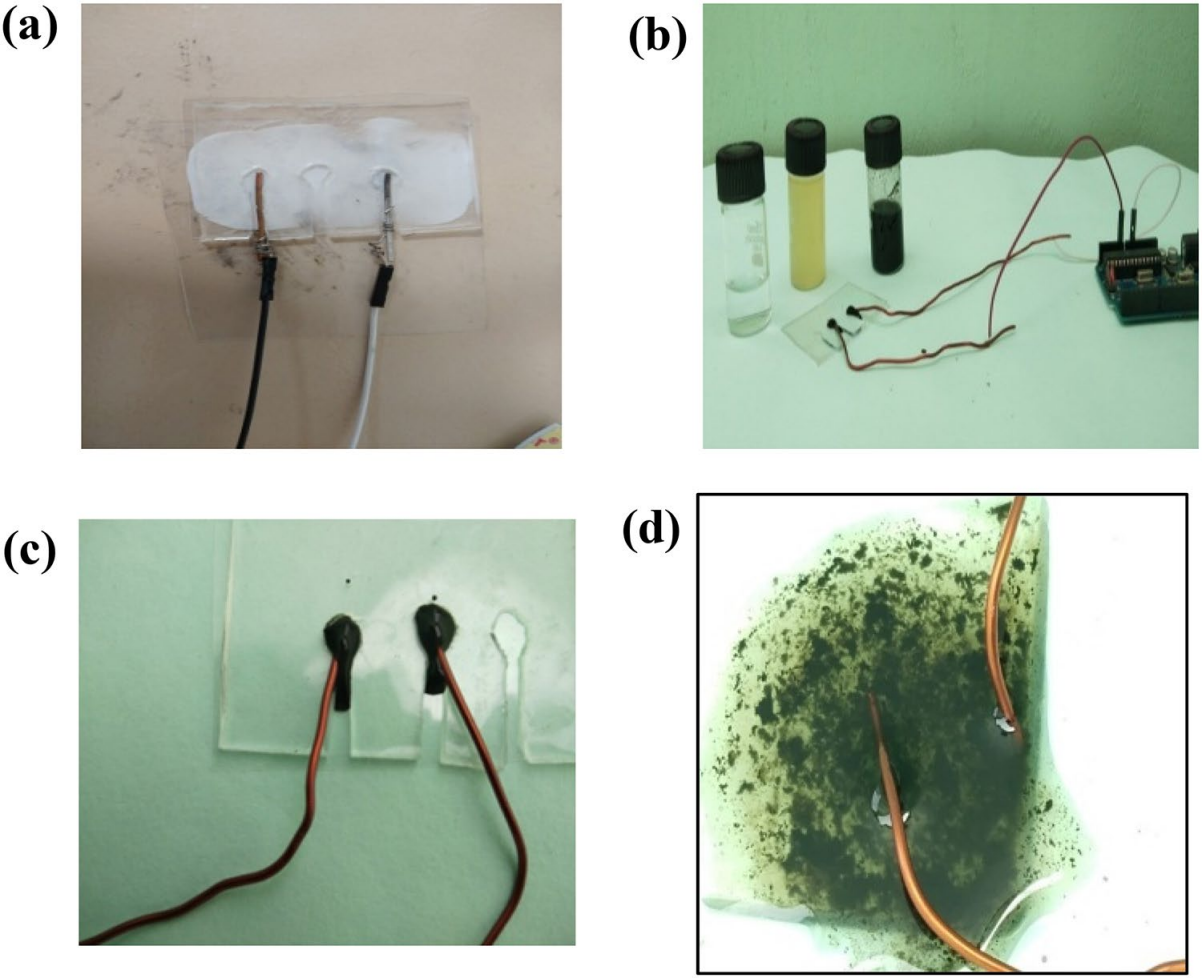
Experimental setup comprising of the following: (**a**) Hydrophobic testing of the sensor base; (**b**) PC-based analysis of normal water samples; (**c**) Voltage measurements with Cu electrodes; (**d**) *E. coli* samples inoculated in *f*GO solution.

The bacterial count was determined and the optical density was also measured. The real-time implementation of the work could be done only after proper offline studies and analysis. The experiment setup consists of the PDMS-based nanosensor; Copper wire (1 mm thickness) was used as working electrode, data acquisition using ATMEGA 328 development board and computer for interpretation and analysis. Since the majority of the samples were contaminated water samples, it was necessary to study the hydrophobic nature of the sensor developed^28^. If the sensor was found to be hydrophobic the tendency to clog between the surfaces prevails, which makes the sensor less effective. The hydrophobic test was analyzed and was shown in Fig. 4a. The voltage measurement using a microcontroller was depicted in Fig. 4b. The figure also shows the distilled water sample, *E. coli* sample, and GO-based nanosensor solution in glass tubes. Figure 4c,d represent the GO before and after mixing with *E. coli* samples. The efficiency of the sensor was tested using the real water sample from the lakes in the region of interest, which eliminate the process of serial dilution. The collected samples were analyzed for the determination of bacterial count using the spread plate technique, and the impedance measurements were performed in parallel to the contamination count.

The chemical reactions of the bacteria with the nanomaterial were analysed^29^. It was observed that the dilution of the bacterial solution with *f*GO solution has resulted in an interlocking through van der Waals interactions. The change in voltage was due to the change in the conductivity of the developed *f*GO nanosheets when interacted with *E. coli*. This is in fact due to the change in impedance offered by the *f*GO nanosheets. An efficient method to measure impedance is necessary at this juncture. A simple cost-effective impedance analyzer made of AD5933 was used. The low power consumption and other exemplary features of AD5933, when compared to standard impedance spectroscopic measurement, make it more reliable to use for data monitoring. The inbuilt frequency generator is used to excite frequencies through the electrodes. The output current signal was fed to a trans-impedance amplifier to read the output in voltage. These voltage values were read by the processor and were sampled at each frequency of excitation. The stored sample data was analyzed and sent to the microprocessor through the I2C interface^30^. It was observed that the impedance was unknown for few frequency ranges, though the output currents were produced.

The limitations of the above method, the analyzer circuit were modified by adding a high impedance application-based amplifier, viz*,* AD8646. This change facilitates the reductionof the cycling effects and also enhances the frequency range of measurement. Two impedance analyzer chips were used to measure the voltage across the unknown impedance and the current. To aid the AD8646 amplifiers were used. Depending on the frequency produced by the impedance analyzer, voltage is produced. The magnitude coefficients of the output were sent to the second analyzer, which measures these magnitude components proportional to the current. An external clock pulse was also used to synchronize data acquisition between two impedance analyzers.

The experimental results were outlined in Table 1. In the initial stages of the experiment, different bacterial samples were taken from the *E. coli* cultured nutrient agar medium^31^. The amine-functionalized GO nanosheets dispersed mixture was kept at a constant value (0.6 mL). For easy analysis, 0.2 mL of bacteria sample was inoculated to *f*GO solution and the procedure continued till a significant change in voltage was visualized. It was observed that as the bacterial concentration increases, the step value also increases, increasing voltage.

**Table 1:** Experimental data for various bacterial concentrations.

The concentration of the solution		Step value obtained from ADC of Arduino microcontroller	Voltage acquired in multimeter (µV)
Sample	Bacterial concentration (0.6 mL of *f*GO solution added to *E. coli *solution) ascending (in mL)		
CONC 1	0.2	244	− 0.17
CONC 2	0.4	256	− 0.13
CONC 3	0.6	253	− 0.10
CONC 4	0.9	261	+ 0.01
CONC 5	1.2	261	+ 0.07
CONC 6	1.5	264	+ 0.11
CONC 7	1.7	264	+ 0.17
CONC 8	1.9	264	+ 0.23
CONC 9	2.1	267	+ 0.38
CONC 10	2.3	273	+ 0.44

This interprets that, the *E. coli* antigens were intercalated in the interlayer spacing of *f*GO nanosheets and as it gets locked, the somatic characteristics of the coliforms change and it gets bound towards the *f*GO nanosheets as an interlocking system, in turn changing the electrical conductivity of the graphene sheets. The impedance of the sensor increases, which results in a high potential drop across the bonds and thus an increase in voltage.The initial readings were taken from distilled water and normal tap water. The polarity of distilled water ranges from − 0.10 to − 0.11 and tap water was found to be at − 0.12 to − 0.13. The readings for the GO solution were observed from − 0.14 to − 0.15. All these readingswere in µV.

The experiments were also performed to analyze the sensor voltage at different ratios of *f*GO nanosheets dispersed solution and *E.coli* solution. Table 2 represents the various analyze done for different weight equivalent ratios of *f*GO dispersed solution to *E. coli* sample. The measurement was restricted to less sample content (less than 4 mL) due to the limitations in the determination of the optical density of the sample under test. The bacterial concentrations from the plate method and deriving from optical density were identical. A correlation was made to the step change (Voltage data was not steady and might result in false reading) to the bacterial concentration. The least concentration sample was counted to have a concentration of 600 × 10^8^ CFU/mL.

**Table 2:** Experimental data for varying molar weight of antigen and antibody solutions.

Distilled water (in mL)	*E. coli *(in mL)	Optical density	Step change in microcontroller	Voltage output (mV)
**Amine functionalized *f*GO based nanosensor solution, 0.05 mL**				
1.5	1.5	1.29	269	− 0.19
2	1	0.9	267	− 0.19
2.5	0.5	0.86	267	− 0.18
2.8	0.2	0.8	266	− 0.17
3	0	0	265	− 0.17
**Amine functionalized GO-based nanosensor solution, 0.1 mL**				
1.5	1.5	0.79	265	− 0.17
2	1	0.65	265	− 0.16
2.5	0.5	0.51	264	− 0.16
2.8	0.2	0.45	262	− 0.15
3	0	0	260	− 0.15
**Amine functionalized GO-based nanosensor solution, 0.5 mL**				
1.5	1.5	0.41	259	− 0.14
2	1	0.37	257	− 0.14
2.5	0.5	0.28	256	− 0.13
2.8	0.2	0.15	255	− 0.13
3	0	0	253	− 0.12

The use of carbon fiber electrodes was used in onsite monitoring resulting in a detection range of 10^3^–10^8^ CFU/mL. Though detection time was less than 5 h, the study focussed on the change in concentration with respect to time and the effectiveness of the sensor to detect the least and maximum time-concentration relation^32^. The higher the contamination, the more difficult it to estimate the count using the spread plate^33^. Also, it was realized that if the volume of GO solution exceeds 1 mL while testing OD, the measurement was null due to the low clearness/turbidity of the water sample. From the above tables, it was identified that there was a decent variation in impedance with the changes in bacterial contamination and the reagent substrate, say, GO solution. A desirable change in voltage is visualized in Table 1. This is due to the high molar fractions of *E. coli* and *f*GO.Though the voltage changes are negligible, the change in step value derived from ADC shows a significant variation in impedance as observed in Tables 2 and 3.

**Table 3:** Experimental data for varying molar weight of *f*GO.

*E. coli* sample 1 mL, distilled water 2 mL		Step value obtained from ADC of Arduino microcontroller	Voltage acquired in multimeter (µV)
Amine functionalized GO-based nanosensor solution (mL)	Optical density		
0	0.06	269	− 0.16
0.1	0.07	265	− 0.15
0.2	0.19	268	− 0.15
0.3	0.28	262	− 0.14
0.4	0.47	260	− 0.13
0.5	0.64	262	− 0.13
0.6	0.76	257	− 0.11
0.7	1.06	256	− 0.09
0.8	1.34	255	− 0.08
0.9	1.76	252	− 0.07
1	> 2	251	− 0.08

However, the primary objective of the study was to develop a sensor for the analysisof the contamination extremities in a complex water matrix. Though the prepared water samples were gathered from the foothills of the Siruvani River, there were other predominant contaminated water sources within the same geographical area. Tamil Nadu Water Supply and Drainage Board (TWAD) has identified about 449 among 2476 were contaminated sources in the region of the study performed inthe Coimbatore district. A major portion of the water bodies constitutes the wetlands and lakes which reserve water from basins of the Noyyal riveralongside the district. These lakes have a major role in maintaining the ecosystem of the region. Literature depicts that majority of these lakes were the primary source of water for the urban populated cities and are highly polluted. Hence continuous monitoring of these sources is most needed to ensure safe water consumption. 10 among 24 lakes in Coimbatore were inspected and the Geographical Information System (GIS) represents the location of the lakes identified.

An efficient mapping tool called Geographical Information System (GIS) was used to illustrate the location of lakes identified for *E. coli* detection (see Supplementary Fig. S14). It was noteworthy to report that all the lakes identified were either developed or naturally occurred and filled up due to the climatic and human interventions. Most of these water bodies were located within the heart and the outskirts of the city and were the primary source of water for the urban population and industries.Hence, there arises a need for quality monitoring of these water sources. Conventional quality testing comprises of testing the basic water parameters like pH, turbidity, and Total Dissolved Solvents which were comparatively easy to detect. As stated above, *E. coli* detection was time-consuming and the effect of contamination affects the victims more rapidly. Hence, an effective system also facilitates to address the problems associated with water source which was commonly utilized by the inhabitants.

The water samples were analyzed at the lake site for impedance change and voltage detection. The same was carried to the laboratory to analyze the bacterial count and to calibrate with the colorimeter to check the optical density. The procedure and methods were similar to the experiments conducted for developed *E. coli* samples using known strains (see Supplementary Fig. S17). As illustrated in Tables 1, 2 and 3, known *E. coli* samples with varying concentration was used to study the effectiveness of the sensor. The efficiency of the sensor was also tested using the real water samples and the results were depicted in Table 4. As the samples were collected from a different geographical region, the orderly severity of contamination cannot be shown. However, the laboratory investigations prove that each of the identified lakes was contaminated in various levels in the range of less than 200 × 10^2^ CFU/mL.

**Table 4:** Experimental data for a real water sample and using developed nanosensor—laboratory analysis.

Sample number	Optical density	*E. coli* count using agar plate	*E. coli* sample 0.5 mL mixed with 0.5 mL of functionalized GO solution		
			Optical density	Step value obtained from ADC of Arduino microcontroller	Voltage acquired in multimeter (µV)
1	0.46	140	1.93	19	0.0927
2	0.51	182	1.62	25	0.1220
3	0.34	187	1.79	21	0.1025
4	0.47	182	1.89	26	0.1269
5	0.53	130	1.92	21	0.1025
6	0.62	111	1.87	24	0.1171
7	0.53	123	1.89	23	0.1123
8	0.47	194	1.94	24	0.1171
9	0.39	183	1.79	22	0.1074
10	0.51	173	1.46	26	0.1269

On analysis, it may be interpreted that the known *E. coli* samples possess an impedance variation in accordance with the concentration of the bacteria. Whereas, the real sample collected from the water bodies also exhibits a change in impedance as clearly depicted in the table, a change in voltage and optical density does exist. This can be even more proved by understanding Table 4. The idea of using functionalized GO was to link the bonding of *E. coli* bacteria, hence no other bacterial source or contaminants can give way to the rise of potential or density or a mere change in impedance. Hence the method can be effectively used in *E. coli* detection in less time. However, the application of artificial intelligencein the future can even make the sensor more efficient so that the *E. coli* count and impedance change can be more interrelated using a trained set of data through an appropriate learning strategy.

## Conclusions

In this work, a unique class of intercalated graphene-based nanosensors was developed, to detect the *E. coli* at very low concentrations in the complex water matrices. For the experimental studies, the bacterial count (CFU) of synthesized *E. coli* samples and real water samples were counted and analyzed. The temperature variations and other unpredictable factors may affect the physical and chemical properties of the bacteria under test. Hence, there was a need of developing a technique to measure the coliforms content in the real-time analysis platform to access the impact on the aquatic environment. The device fabrication was executed with the PDMS polymeric substrate with microfluidic channels for the electrodes. When the sensor contacts the aquatic environment along with the increase of *E. coli* bacterial contamination, the output voltage was also increased. This led to the development of rapid detection of *E. coli* bacteria using nanosensors by the fabrication of amine-functionalized GO nanosheets. These nanosheets facilitate the larger interlayer *d*-spacing for the selective grafting of *E. coli* in low concentration detection. Among the sensors available in the market, the majority are expensive and bulky. To overcome these limitations, a nanosensor was developed with high efficiency and rapid detection of bacterial concentration. Though the development of one such sensor is costly, mass production utilizing 3D printed polymers helps in commercializing the product and its usefulness to a great extent.

## Experimental

### Materials and methods

The samples of known concentrations of *E. coli* contaminated water were prepared using the serial dilution method. The microbial count of these samples is determined using Spread Plate Method. These samples were inoculated to the functionalized GO nanomaterial in various molar ratios, and the voltage values were noted. For ensuring better calibration, the optical densities of these samples were also measured simultaneously. The water samples from various water matrices were collected and tested using the developed sensor for real water analysis. Conc. H_2_SO_4_, sodium meta periodate, ethanol, tetrahydrofuran, H_2_O_2_ (30%), Diethylenetriamine (DETA), and phthalic anhydride were purchased from Sigma-Aldrich. Polydimethylsiloxane (PDMS) is a silicone-based organic polymer used for the fabrication of nanosensors^34^. The silicone elastomer curing agent and its elastomer base were purchased from Sigma Aldrich. The protected DETA functionalized GO and free amine-functionalized GO (*f*GO) were prepared by the previously reported procedure^35^.

### Preparation of bacteria culture

The strains of *E. coli* Shiga toxins were cultured in a liquid broth medium and inoculated to develop in higher concentration^31^. The Heart-Brain Infusion (HBI) Broth was taken to culture *E. coli* samples. The standard laboratory preparation method^36^ was followed to culture the bacteria in the general-purpose HBI medium. The constituents of the medium were rich in carbon, nitrogen, and essential amino acids and vitamins enhancing the growth. In the media, dextrose, sodium chloride, and disodium phosphate act as the source of energy, equilibrium balancing, and buffering agents, respectively.

The procedure for the preparation of the broth was as follows: suspend 37.0 g of nutrient medium powder in 1 L of deionized water. Stir the solution until the medium dissolves completely. After dispensing the required quantity, the mixture was placed in an autoclave at 121 °C for 15 min. The prepared culture medium is stored at a very low temperature in a sealed container. This is the procedure of growing *E. coli* in the culture medium (BHI broth): The prepared medium was allowed to acclimatize to the room temperature, further it was inoculated using O157:H17 strains. The medium was incubated in closed containers for preventing external contaminants and the growth was examined after 48 h. After the incubation period tubes were examined for turbidity which was an indication of the growth. Later, the culture broth was sub-cultured to various test samples in different concentrations and the colonies were observed (Fig. 2) by Pour Plate Method.

### Preparation of bacterial samples by serial dilution method

The experiment was performed with ten samples with varying *E. coli* concentrations and the sample solutions were prepared using serial dilution which involves the process of mixing the known amount of sample to deionized water resulting in one concentration^37^. 1 mL of prepared *E. coli* medium was inoculated to 9 mL of distilled water, resulting in the sample with the highest concentration. 1 mL from the new sample mixture was inoculated to 9 mL of distilled water to give 1/10th molar concentration of the parent sample. The process was repeated several times to obtain the number of samples with various concentrations. The dilution factor of 1:10 or 10^–1^ was added after each dilution, and thereby reducing the concentration. The known volumes of these samples were spread to the nutrient medium, and by identifying the number of colonies formed, the microbiological count was determined.

### Spread plate method

The sub-cultured bacteria samples were poured into the Petri dishes for the bacterial count^38^. The Petri dishes that contain *E. coli* samples at various concentrations were incubated at a warm temperature between (35–37 °C) for 48 h or more, but not more than 70 h (more time-lapse will enhance the bacterial growth in Petri dish). The number of visible bacterial colonies was counted either using markers or using manual counting for a square area using a scale marker. The number of the viable bacterial cell was measured as colony formation unit (CFU) was expressed as.

1 $$ {\text{Bacteria }}\left( {{\text{CFU}}} \right)/{\text{mL}}\, = \,\left( {{\text{no}}. \, \,{\text{of}}\,{\text{ colonies }} \times {\text{ dilution }}\,{\text{factor}}} \right)/{\text{volume }}\,{\text{of }}\,{\text{culture}}\,{\text{ plate}}{.} $$

Thereby, the bacterial concentration in CFU was estimated. The collected water samples were also analyzed using the standardized technique.

### Development of PDMS substrate

The process was executed with the blending of PDMS, elastomer base, and elastomer curing agent (thickener) in a ratio of 10:1. The proper mixing was ensured by using a head stirrer with a nominal speed of about 100 rpm until the thickener and base material dissolves perfectly. After the dilution of the curing agent to the base elastomer, a thick solution full of foam was obtained which should be desiccated for removing the air bubbles. A vacuum desiccator was used at normal pressure of about 500–600 mmHg. After few minutes the vacuum inside the desiccator was liberated which also helps in popping the bubbles from the mixture. The procedure is repeated until to get a clear solution.

A 10 cm × 4 cm glass slide was used as the base for the PDMS substrate. The hydrophobic nature of the PDMS base tends to repel the water containing the bacterial content. Hence to hold the sensing material and the sample, microfluidic channels have to be imparted before pouring onto the glass plate similar to most of the wearable nanosensors and biomaterials that were developed^39^. The general purpose adhesive tape was mounted on the sides of the glass plate to restrict the flow of the solution within the glass boundary. Three thin glass strips (1 mm) were kept horizontally to the glass slide for the microfluidic space as illustrated in Fig. 3 (Stage 3). The desiccated solution was poured into the glass slide. The bubbles were removed completely and the resulting mixture is left to sit overnight to solidify. The polymer was then baked in an oven at 70–90 °C for 1 h. The polymer was allowed to cool at room temperature for a couple of hours after baking using a knife and a ruler. The edges of the adhesive tape were cut and the PDMS was peeled off. The synthesized PDMS substrate was attached to a self-adhesive polymer or plastic sheet so that the top layer shows the PDMS microfluidic channel and the bottom layer would be the polymer to support the sensor material.

### Synthesis of amine-functionalized graphene oxide-based 2D nanomaterial

#### Synthesis of graphene oxide

Graphene Oxide was prepared by the reported method^40^ from graphite powder. In a typical reaction, to the stirred solution of 1 g of graphite powder in 40 mL of Conc. H_2_SO_4_ (98%), 12 g of sodium meta periodate was added at ice-cold temperature with vigorous stirring for 24 h. The thick, grey color paste was then diluted slowly by the addition of 150 mL of distilled water with continuous stirring to get the color change from grey to dark brown color. To this mixture, 5 mL of H_2_O_2_ (30%) was added and stirred for 1 h with the addition of Conc. HCl (5 mL). The brown dispersed solution was centrifuged and the brown solid GO was washed with 1:1 (*v/v*) aqueous ethanol (3 × 20 mL) to remove the water and diethyl ether (20 mL) to get the free flow solid. The dark brown product was dried and stored in the desiccator.

### Preparation of amine-functionalized GO-DETA nanosheets

The DETA functionalized GO was prepared via three simple steps. *Step 1* Phthalic anhydride (5.7 g, 0.038 M) was heated to 120 °C for 30 min with constant stirring, and Diethylenetriamine (2.1 mL, 0.019 M) was slowly added to the molten state. The dense white fumes were condensed into the reaction flask. To this residue, ethanol (100 mL) was added and stirred for 2 h. Upon cooling, the bis(phthalimidoethyl)-amine was formed as pale a yellow solid. It was used for the further process without purification. Yield: 94% (6.8 g). *Step 2*: The protected amine, bis(phthalimidoethyl)-amine was used to intercalate the GO basal planes. In a typical reaction, the well-dispersed GO (500 mg) solution in 150 mL of de-ionized water was sonicated for 30 min. To this dispersed solution, Bis(PIEA) amine (1 g) in ethanol (40 mL) was added dropwise under continuous stirring for 24 h. Finally, the mixture was centrifuged at 5000 rpm for 10 min to isolate the GO/bis(PIEA). The GO/bis(PIEA) solid was washed with ethanol and diethyl ether to isolate the free-flow black solid. *Step 3:* The protecting group phthalic acid was removed by the acid treatment. Typically, GO-bis(PIEA) (300 mg) was stirred with 10% of HCl solution under RT condition for 24 h. The GO-DETA was isolated by centrifugation of the above mixture at 5000 rpm for 10 min. The resultant free amine DETA functionalized GO was washed well with hot water, ethanol, and diethyl ether to isolate the black solid. The sensor solution was made by mixing 20 mg of amine-functionalized GO material with 10 mL of conductivity/HPLC water. The mixture after sonication for about 15 min was ready for use. The intercalated materials GO-Bis(PIEA) and GO-DETA were characterized using XRD, FT-IR, XPS, RAMAN, and TEM (refer Supplementary Figs. S2–S6) for the confirmation of functionaliztionations.

## Supplementary Information

Supplementary Information.
