# Supplementary material for: Polyaza functionalized graphene oxide nanomaterial based sensor for Escherichia coli detection in water matrices
Source: Sci Rep. 2021 Aug 19;11:16872. doi: 10.1038/s41598-021-96539-6 (PMC8377045; doi:10.1038/s41598-021-96539-6)
Supplement: Supplementary file 1 — Supplementary Information. [file 41598_2021_96539_MOESM1_ESM.docx]

Polyaza Functionalized Graphene Oxide Nanomaterial Based Sensor for Escherichia Coli Detection in Water Matrices

Lina Rose,^1^Anitha Mary X,^2^ Johnson I,^3^ Ganesh Srinivasan,^4^Lakshmi Priya,^4^Jebasingh Bhagavathsingh^4^*

^1^Department of Biomedical Engineering, Karunya Institute of Technology and Sciences, Coimbatore-641 114, Tamil Nadu, India.

^2^Department of Robotics Engineering, Karunya Institute of Technology and Sciences, Coimbatore-641 114, Tamil Nadu, India.

^3^Department of Millets, Tamil Nadu Agricultural University, Coimbatore - 641 003, Tamil Nadu, India,

^4^Department of Applied Chemistry, Karunya Institute of Technology and Sciences, Coimbatore-641 114, Tamil Nadu, India.

Correspondence: [jebasinghb@karunya.edu](mailto:jebasinghb@karunya.edu) ; Tel : +91-422-2614409, Fax: +91-422-2615615.

**Electronic Supporting Informations (ESI)**

| **S. No** | **Contents** | **Page No.** |
| --- | --- | --- |
| 1. | Impedance Measurement using Arduino Uno Board | S3 |
| 2. | Figure S1. Measurement using Arduino- Schematic | S3 |
| 3. | Program | S3 |
| 4. | **Characterizations of Intercalated GO materials** | S5 |
|  | Figure S2. PXRD spectra of GO, GO-Bis(PIEA) and GO-DETA | S5 |
|  | Figure S3. FT-IR spectra of GO, GO-Bis(PIEA) and GO-DETA | S5 |
|  | Figure S4. TEM Images of GO, GO-Bis(PIEA) and GO-DETA | S6 |
|  | Figure S5. Raman Spectra of GO, GO-Bis(PIEA) and GO-DETA | S6 |
|  | Figure S6. XPS Spectra of GO- GO-Bis(PIEA) and GO-DETA | S7 |
|  | Table S1. Elemental Analysis of the Intercalated Materials | S8 |
| 5. | **Measurement using Impedance Analyser** | S8 |
|  | Figure S7.AD5933 Functional Block Diagram | S8 |
|  | FigureS8. Experimental values at different frequency | S9 |
|  | Figure S9. Block Diagram of Impedance Analyser. | S9 |
|  | Figure S10**.** a) Cultured *E.coli* sample, b) *E.coli* developed in Petri dishes, c) Bacterial counts using serial dilution plating. | S10 |
| 6. | **Experimental Setup** | S10 |
|  | Figure S11. Prepared bacterial sample using serial dilution | S10 |
|  | Figure S12. Optical Density measurement setup | S10 |
| 7. | **Representation of proposed mechanism** | S11 |
| 8. | The geographical areas identified as complex water matrices | S12 |
| 9. | **List of Lakes in Coimbatore City** | S12 |
| 10. | **GIS location of various lakes identified** | S13 |
|  | Table S2. GIS data of the lakes identifies for collection of samples. | S13 |
|  | Figure S13: Geographical illustration of the lakes using a GIS platform. | S13 |
| 11. | **Sample Collection and Analysis** | S14 |
|  | Figure S14. The sample collection and Voltage detection of the sample at lake site | S14 |
|  | Figure S15. Testing of Samples collected from lakes | S14 |
|  | Figure S16: Experiment analysis as follows a)collected water samples after incubation for 24 hours, b) bacterial count in CFU using agar plate technique. | S14 |
| 12. | **References** | S14 |

**1. Impedance measurement using Arduino Uno Board**

The copper electrodes were connected to the analog pin of the microcontroller which reads the analog voltage. The inbuilt analog to digital converter in the controller, produces step changes for each analog voltage value, which in turn was an indication of the concentration change. The bacterial count was determined and the optical density was also measured. The real time implementation of the work could be done only after proper offline studies and analysis. The experiment setup consists of the PDMS based nanosensor; Copper nanowire is used as working electrodes, data acquisition using ATMEGA 328 development board and computer for interpretation and analysis.The simplest way to measure an analog voltage is using an Adruino software (IDE). The board uses an ATmega 328 microcontroller as a main processor and has 6 inbuilt Analog to digital converters. When exposed to various impedances, there develops a potential drop across the Cu wires which is connected to Analog Voltage input and ground terminals of the board. The schematic gives a clear understanding of the data acquisition.


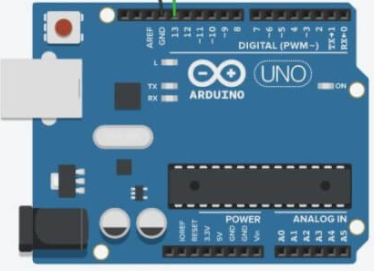


Serial Monitor

**FigureS1**. Measurement using Arduino- Schematic

**2. Program:**

// Connect the Analog input of Turbidity sensor1 A0

#include <SoftwareSerial.h>

SoftwareSerialBTserial(10, 11);

int sensorPin1 = A0;

void setup()

{

// Initialize Bluetooth, set the baud rate to 9600 bps.

BTserial.begin(9600);

pinMode(sensorPin1, INPUT);

// Set pin A5 to use as a power pin for sensor1

Serial.begin(9600);

}

void loop()

{

// Variable to store voltage1 calculation

float voltage1;

float voltage3;

float voltage4;

float voltage5;

// Read the raw 0-1023 values of voltage into a variable.

rawvolt = analogRead(sensorPin1);

// Calculate the voltage, based on that value.

// Multiply by maximum voltage (5V) and divide by maximum ADC value (1023).

voltage1 = rawvolt * (5 / 1023.0);

Serial.print("voltage1: ");

// Print voltage1 reading to serial monitor

Serial.println(voltage1);

Serial.println();

// Wait 1 second between readings

delay(1000);

BTserial.print(voltage1);

BTserial.print(";");

//message to the receiving device

delay(200);

}

**4. Characterization of Intercalated GO materials (XRD,FT-IR, TEM, Raman and XPS)**


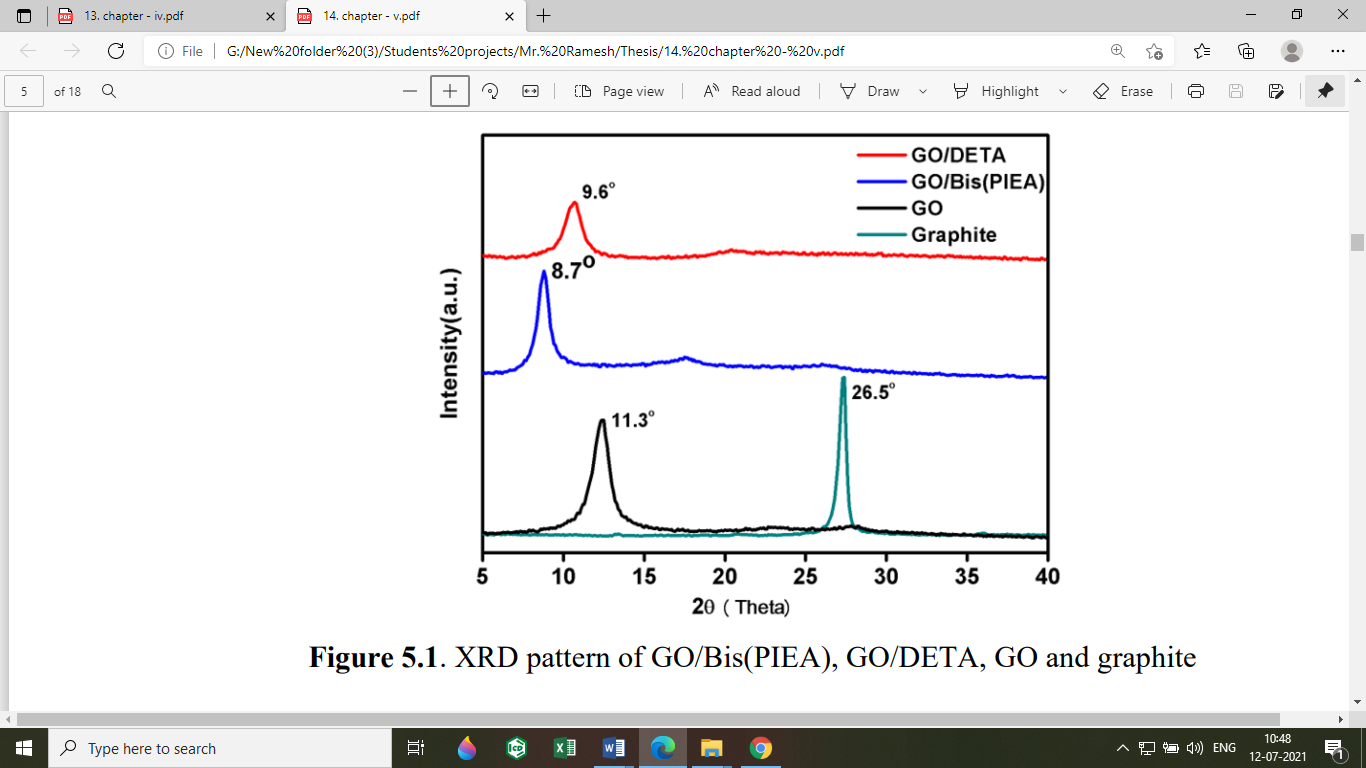


Figure S2: PXRD-Patterns of GO, GO-Bis(PIEA), GO-DETA


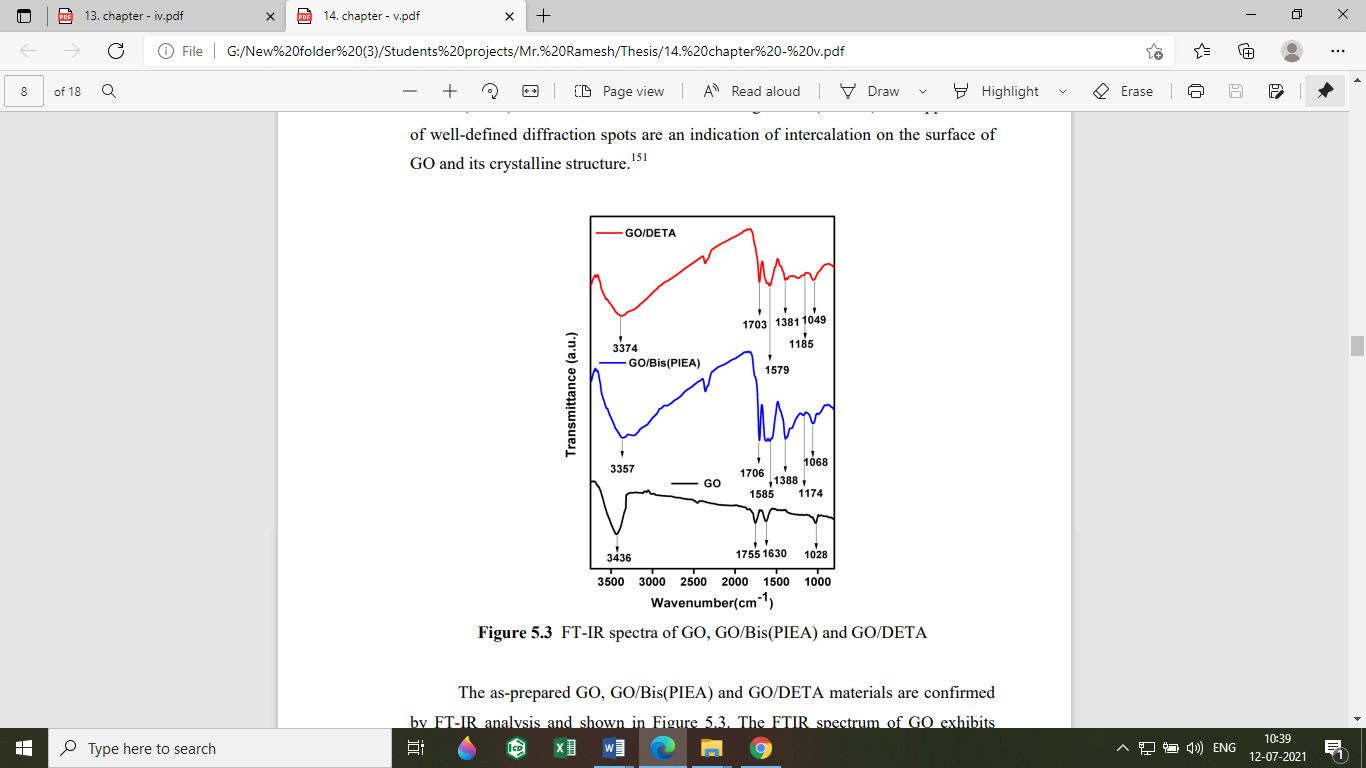


Figure S3: FT-IR Spectra of GO, GO-Bis(PIEA), GO-DETA.


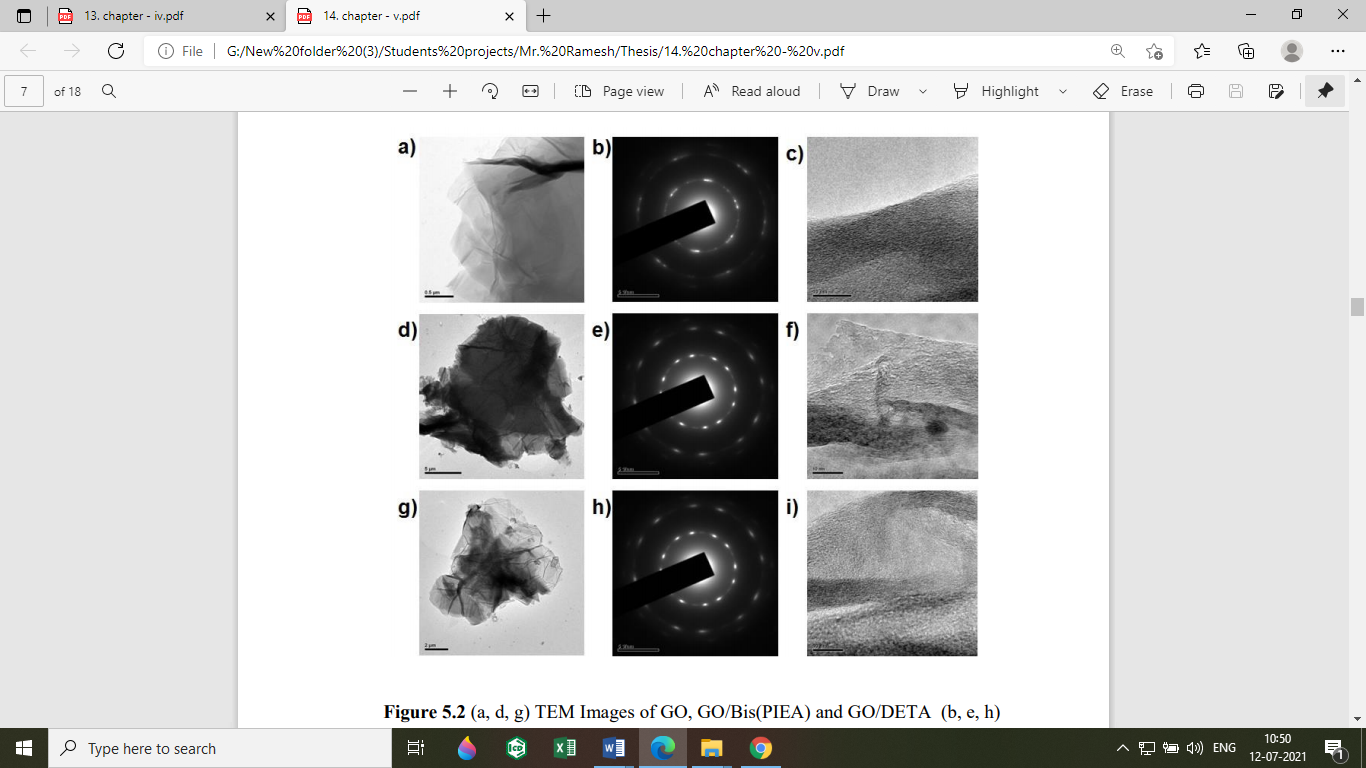


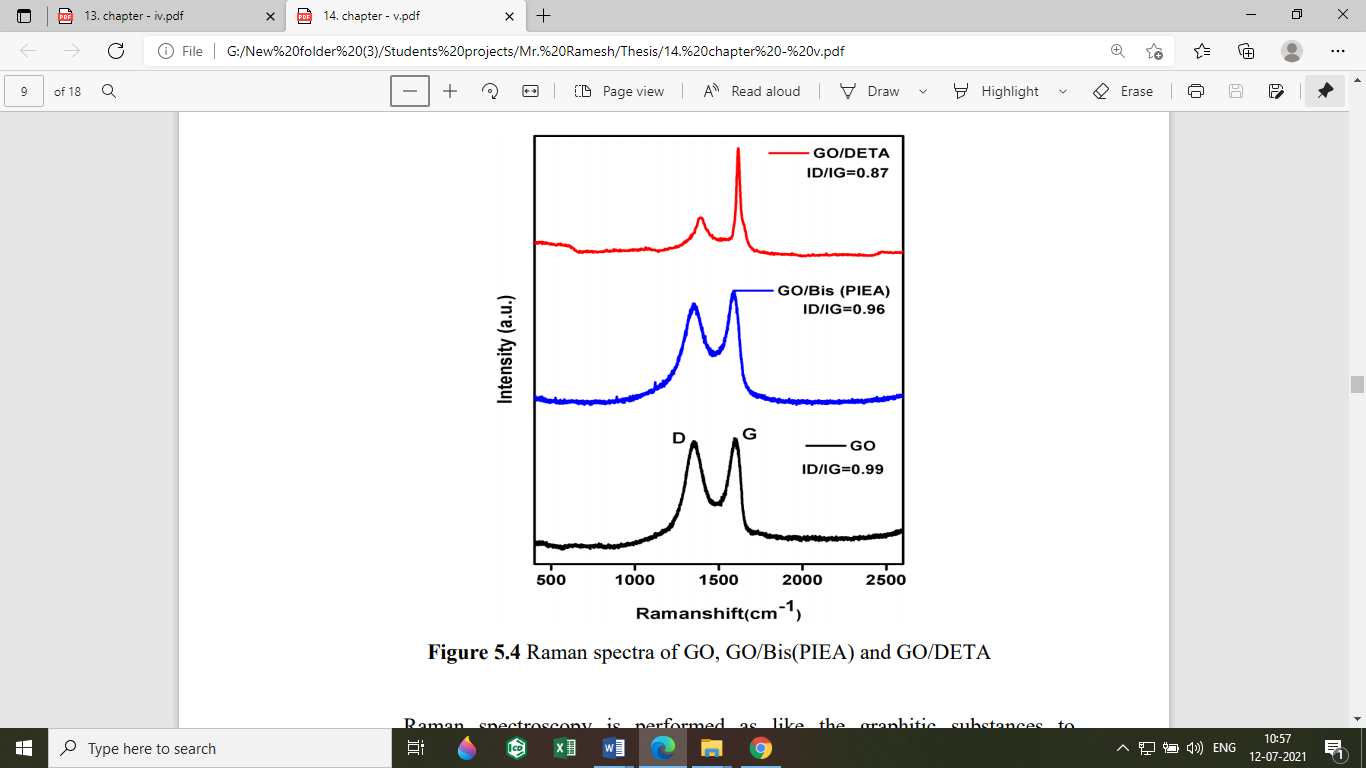
Figure S4: TEM Images of GO, GO/Bis(PIEA) and GO/DETA (a, d, g) SAED pattern image of GO, GO/Bis(PIEA) and GO/DETA(b, e, h) HR-TEM images of GO, GO/Bis(PIEA) and GO/DETA (c, f, i ).

Figure S5: Raman spectra of GO, GO-Bis(PIEA), GO-DETA


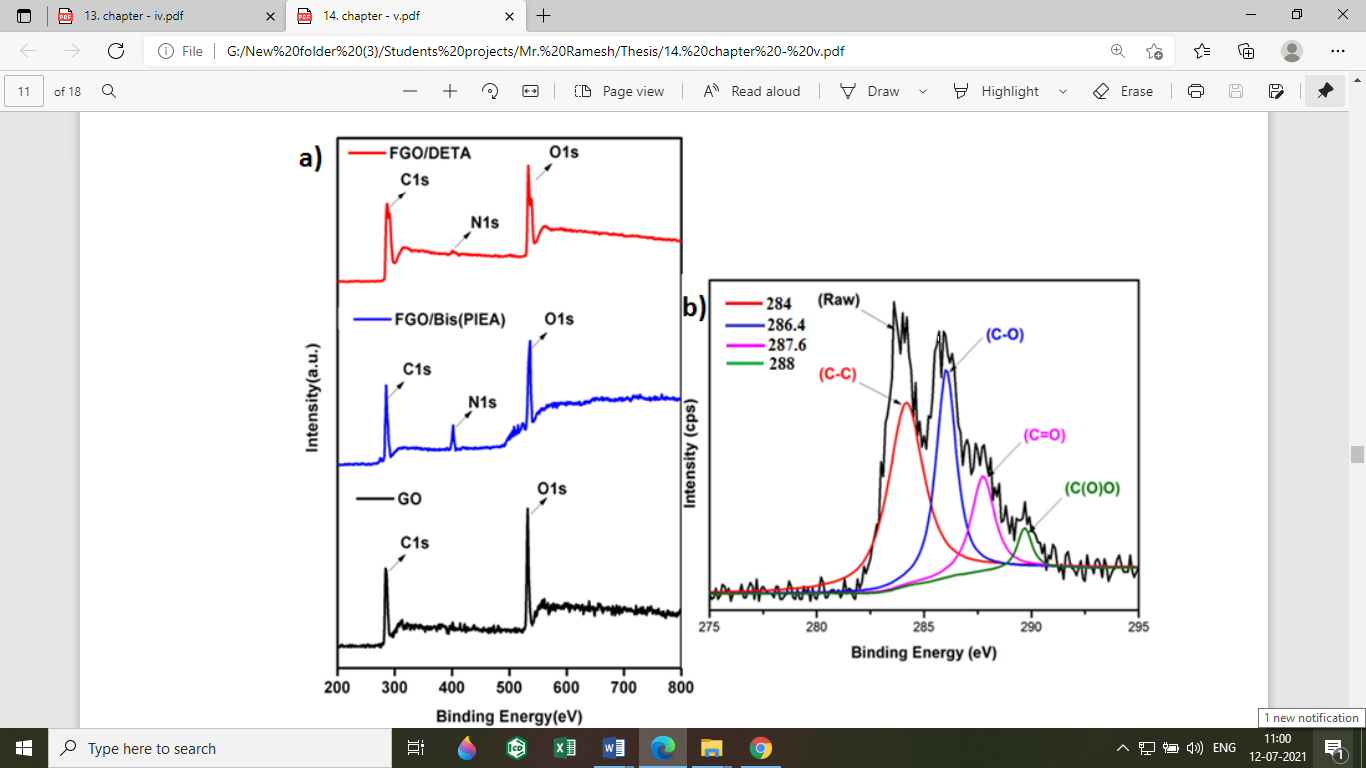


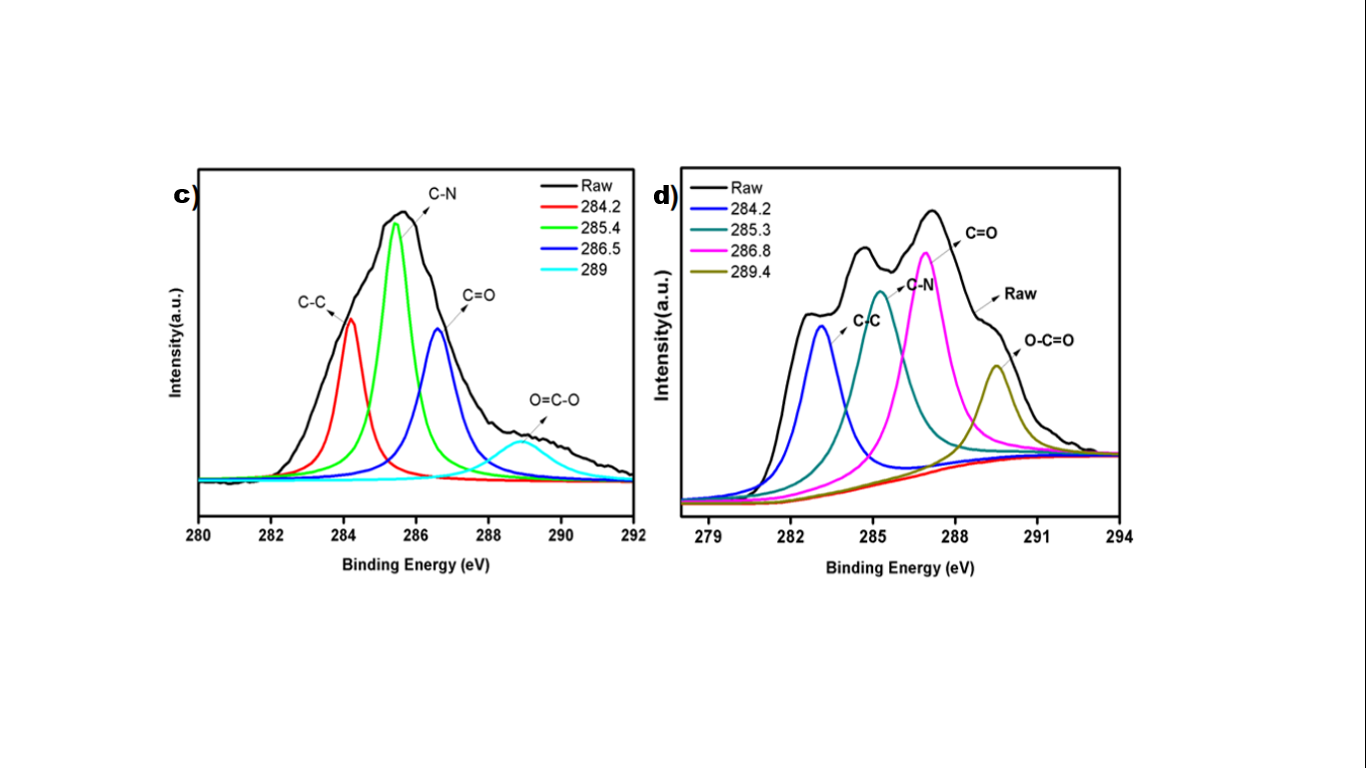


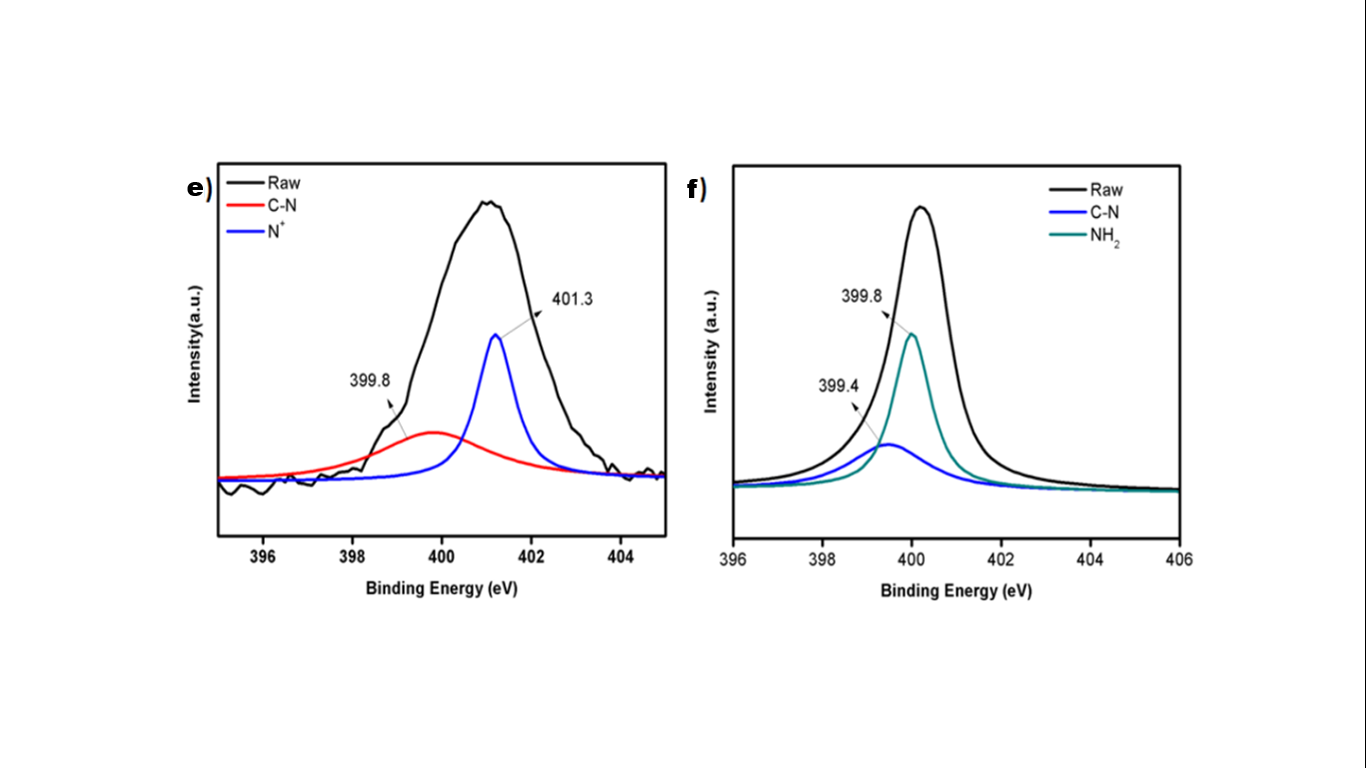


Figure S6: XPS spectra (a) full spectrum of GO, GO/Bis(PIEA) and GO/DETA, (b) XPS for deconvoluted C1s GO, (c and d) XPS for deconvoluted C1s GO/Bis(PIEA) and GO/DETA, (e and f) XPS for deconvoluted N1s GO/Bis(PIEA) and GO/DETA.

**Table S1:**EDX Elemental Analysis of the Intercalated Materials

| S.No | Materials | C% | O% | N% | S% or Cl% |
| --- | --- | --- | --- | --- | --- |
| 1. | GO | 67.29 | 32.18 | 0.00 | 0.53 |
| 2. | GO-Bis(PIEA) | 63.19 | 33.83 | 2.98 | 0.00 |
| 3. | GO-DETA | 63.67 | 32.89 | 2.82 | 0.62 |

**5. Measurement using Impedance Analyser**

The initial measurement lacks accuracy due to raw impedance measurement. Impedance highly varies with respect to frequency and hence the real and imaginary terms contribute to the electrical impedance as well. Hence an efficient measurement system was needed to analyse the impedance for varying frequencies. AD5933 is such an impedance converter IC, highly precise which has an inbuilt signal generator and ADC (Analog to Digital converter). The on-board frequency generator uses a known frequency to stimulate the external impedance. The ADC samples these impedance values and is processed through an on-board processor, which results in real and imaginary terms of impedance for that frequency response. The resultant real value was read from I^2^C interface.


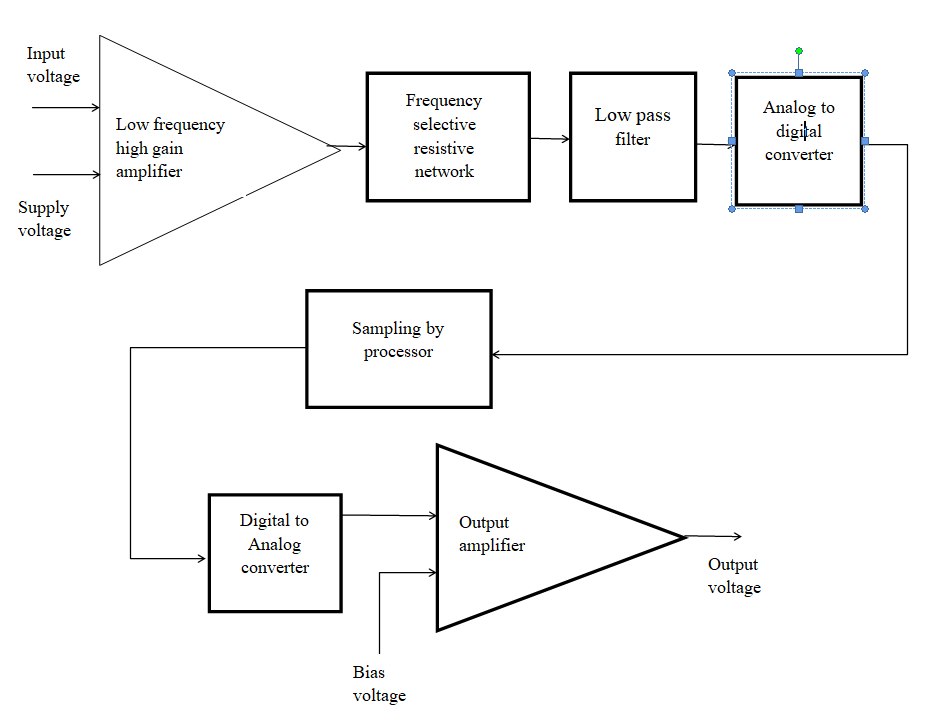


**FigureS7**. AD5933 Functional Block Diagram^S1^

**a)**

**b)**

**FigureS8**. Experimental values at different frequency: a) Frequency response of magnitude component, b) Frequency response of phase component.

The measurement loaded the magnitude errors due to the limitations in repeated calibrations and a wide range of frequencies. Hence, to increase the frequency range an additional AD5933 was incorporated together with a high gain amplifier AD8646.

Differential amplifier (voltage output)

Ai

Av

AD5933

AD5933

CLK

CLK

Microcontroller

Unknown Impedance (Z)

Current to voltage Converter

**Figure S9**. Block Diagram of Impedance Analyser.

The unknown impedance is fed to the two high gain amplifier one acting as a differential amplifier (Av) and the other as a trans-impedance amplifier (Ai). The differential amplifier measures the voltage across the unknown impedance, which is then converted to the impedance excited at various frequencies using first AD5933. At the same instant, the current through the unknown impedance is also measured and is fed to the analyser to measure the real and imaginary components. Both the analysers are operated synchronously through a known clock set by the microcontroller. The measurement method proves to be more efficient in converting impedance values than using a single analyser.

These measurements were performed to ensure that the impedance of the sample varied with different *E. coli* concentration.

**
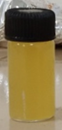

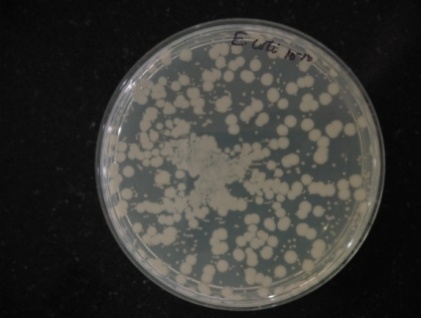

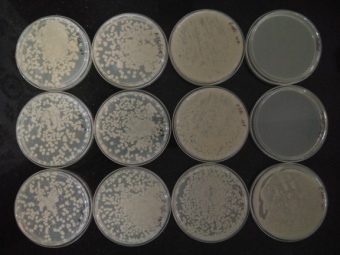
**

1. **(b) (c)**

**Figure S10.** a) Cultured *E.coli* sample, b) *E.coli* developed in Petri dishes, c) Bacterial counts using serial dilution plating.

**6. Experimental Setup**


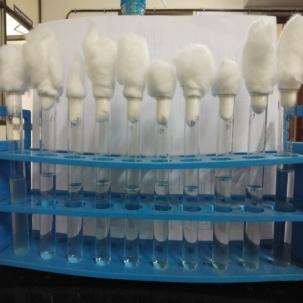


**Figure S11**. *E.coli* Bacterial sample preparation using serial dilution


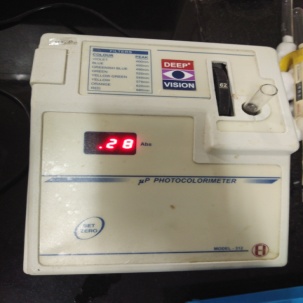


**Figure S12**. OD measurement setup

**7. Representation of Proposed Mechanism**

**PHASE I: Preparation of Escherichia coli samples**

Identification of *E. coli* count in CFU

Pour plate method- for determination of *E. coli* count

Preparation of E coli samples using Serial Dilution

**PHASE III: PC based sensor analysis**

Voltage analysis in PC

Arduino microcontroller

ADC

Impedance amplifier

I

Pour plating

Sample collection

Arduino based voltage analysis

Added to PDMS microfluidic channel having *f*Go

CFU count

GIS based identification of lakes

**PHASE II: Preparation of PDMS substrate**

Sample

Frequency Generation

Impedance change

Microcontroller

**PHASE IV: Analysis using impedance analyzer**

Voltage measurement through Cu electrodes

Solution (*E. coli*+ *f*Go in various molar concentrations) placed in the microfluidic channels of PDMS

Synthesis of PDMS substrate

Preparation of microfluidic structure

Vacuum Desiccation

Material mixing using overhead stirrer

**PHASE V: Analyzing the efficiency of sensor in complex water matrix**

I2C

V

**Figure S13**. The representation of Proposed Mechanism of Detection.

**8. The Geographical Areas Identified As Complex Water Matrices**

Coimbatore is located at [11.0161°N 76.971°E](https://geohack.toolforge.org/geohack.php?pagename=Geography_of_Coimbatore&params=11.0161_N_76.971_E_) in Tamil Nadu state in India. The Western Ghats of the district are the home of rivers such as Bhavani, [Noyyal River](https://en.wikipedia.org/wiki/Noyyal_River), Aliyar, Siruvani which provide the drinking water and irrigation water for the people and farmers of Coimbatore.  Many lakes and ponds were dug around the Coimbatore city in ancient times to reserve the water from Noyyal river (Originates from western ghats). The city of Coimbatore has 24 Lakes (Wetlands ) in the River Noyyal Basin fed by the river. They are also known as System Tanks in Public Works Department (PWD). Singanallur Lake, Kuruchi Lake, Valankulam Lake, Krishnampatti Lake, Muthannan Lake and Seevagasintamani Lake are a few of them. In most of the urban ecosystems, these wetlands are the major life-supporting component with high concentrations of birds, mammals, reptiles, amphibians, fish and invertebrate species.

**9. List of Lakes in Coimbatore City**

- Achankulam
- Anaipalayam Lake
- Andipalayam Lake
- **UkkadamPeriankulam Lake**
- Irugur Lake
- Kannampalayam Lake
- Kathanganni Lake
- Kolarampathy Lake
- **Krishnampathy Lake**
- **Kurichi Lake**
- **Muthannankulam**
- Mudalipalayam
- **Narasampathy Lake**
- Pallapalayam Lake
- **Perur Big Lake**
- **Perur Small Lake**
- **Pudukulam**
- Puttuvikki Lake
- Ramachandra Lake
- Selvachinthamani Lake
- **Selvampathy Lake**
- Senkulam
- Shyamalapuram Lake
- **Singanallur Lake**
- Sulur Big Lake
- Sulur Small Lake
- **Valankulam**
- Vellalore Lake

**10. GIS location of various lakes identified.**

**Table S2.**GIS data of the lakes identifies for collection of samples.

| Sample Number | Location | GIS data |
| --- | --- | --- |
| 1 | MukannamKulam (Kumarasamy lake) | N 11^0^ 00.128^l^  E 076^0^ 56.804^l^ |
| 2 | Perur lake (Perurkulam) | N 10^0^ 58.177^l^  E 0 76^0^ 55. 850^l^ |
| 3 | PerurChetti Lake | N 10^0^ 58.383^l^  E 76^0^ 54.724^l^ |
| 4 | Narasampatti Lake | N 11^0^ 00.214^l^  E 076^0^ 55.253^l^ |
| 5 | UkkadamPeriakulam | N10^0^ 59.099 ^l^  E076^0^ 57.063^I^ |
| 6 | KurichiKulam (lake) | N 10^0^ 58.111^l^  E 076^0^ 58. 056^l^ |
| 7 | Singanallur | N 10^0^ 59.867^l^  E 0 77^0^ 01. 170^l^ |
| 8 | Vadagkulam | N10^0^ 59.712^l^  E 076^0^ 59.031^l^ |
| 9 | Krishnampatty lake | N 11^0^ 00.236^l^  E 076^0^ 55.379^l^ |
| 10 | Selvampatty Lake | N 11^0^ 00.204^l^  E 076^0^ 55.979^l^ |

**
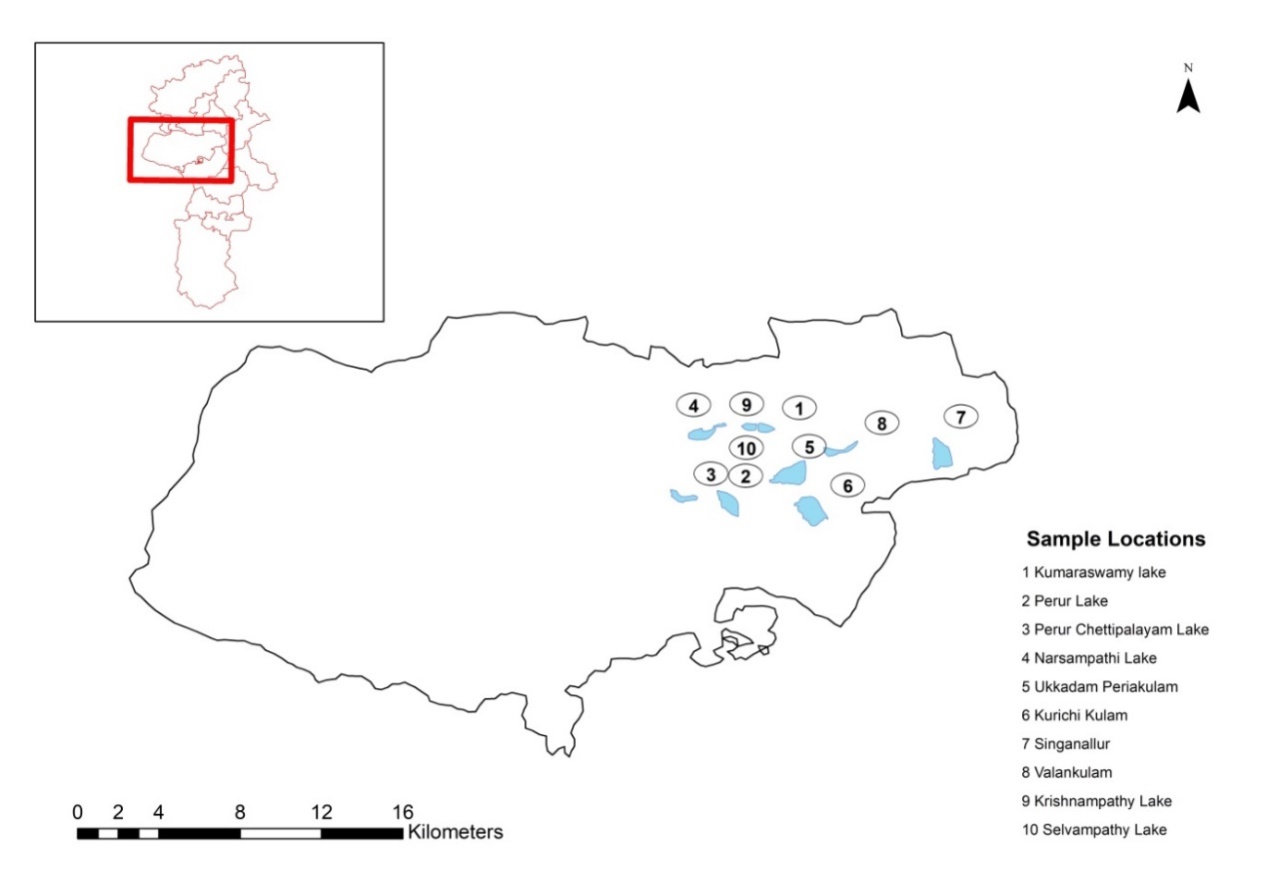
**

**Figure S14**: Geographical illustration of the lakes using a GIS platform.

**11. Sample Collection and Analysis**

**
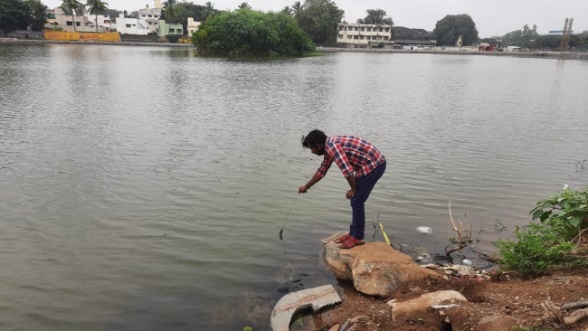

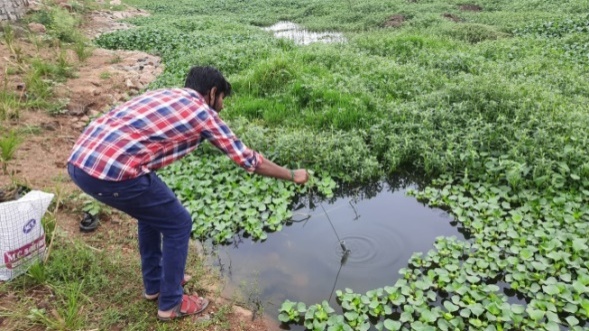
**

**Figure S15**: The sample collection and Voltage detection of the sample at Lake Site
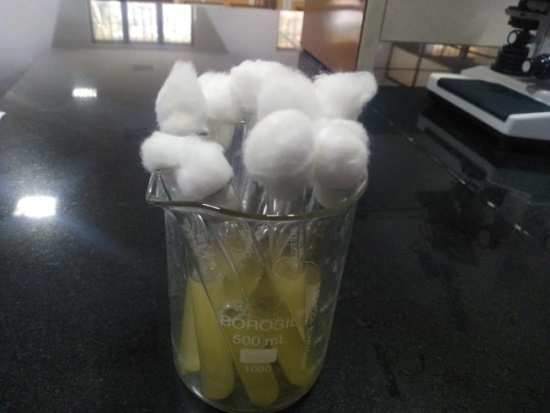

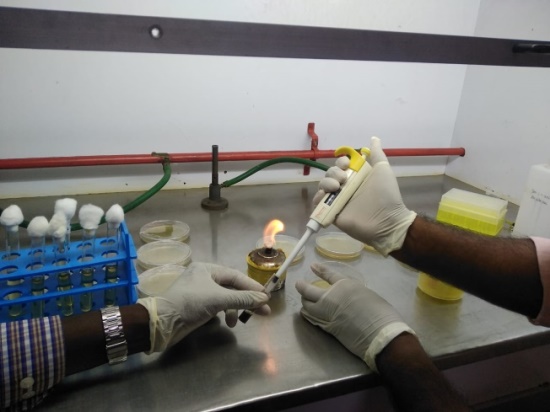


**Figure S16**. Testing of Samples collected from lakes

**
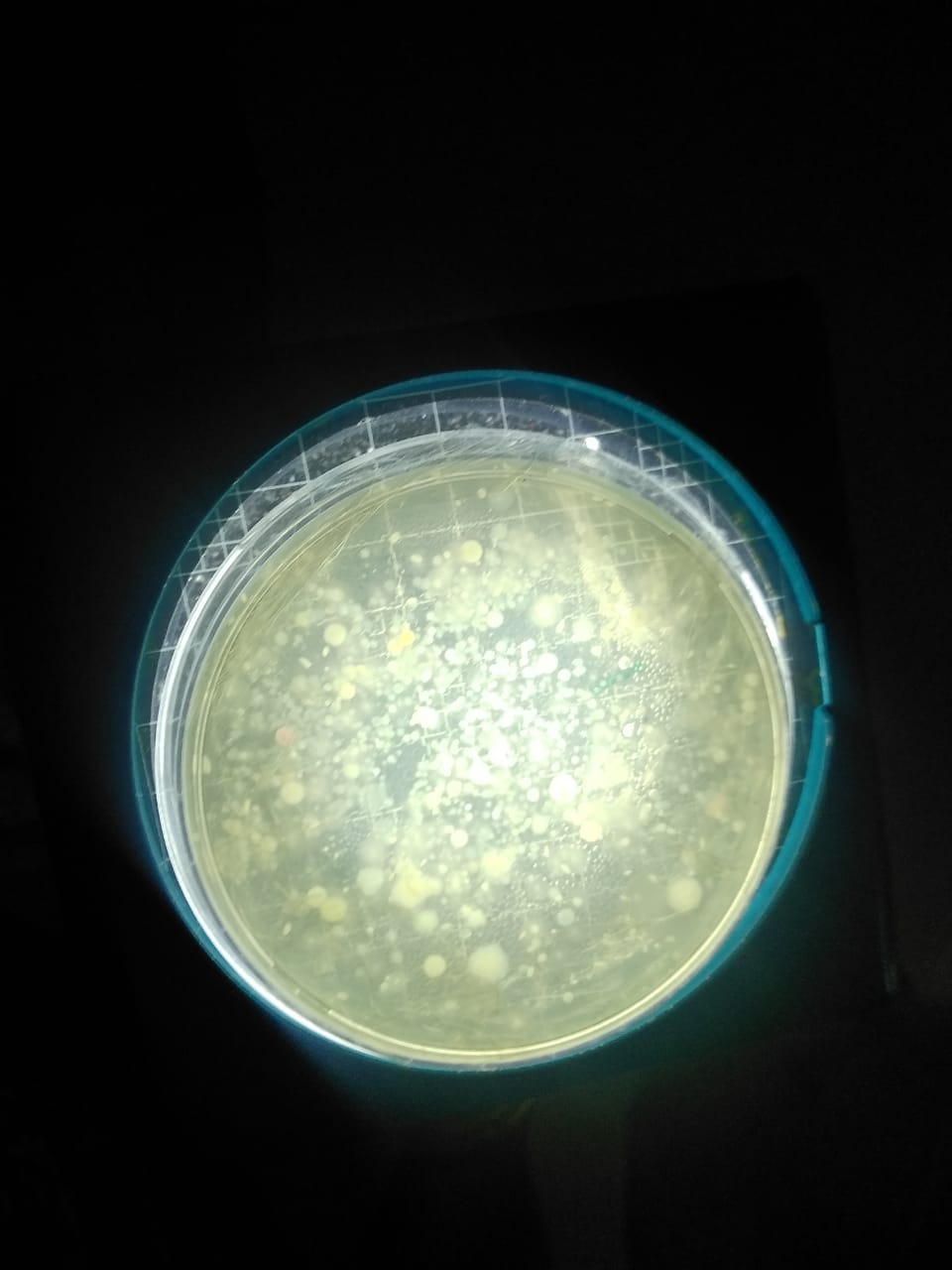

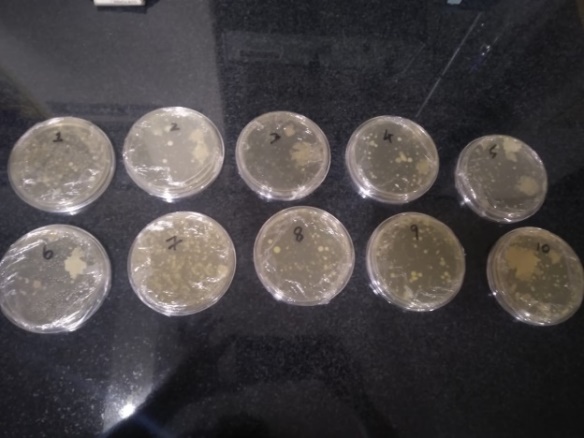
**

**(a) (b)**

**Figure S17.**Experiment analysis as follows a)collected water samples after incubation for 24 hours, b) bacterial count in CFU using agar plate technique.

**12. Reference**:

S1. <https://www.analog.com/media/en/technical-documentation/datasheets/AD5933.pd>f
